# Lassa Virus Circulation in Small Mammal Populations in Bo District, Sierra Leone

**DOI:** 10.3390/biology10010028

**Published:** 2021-01-05

**Authors:** Umaru Bangura, Jacob Buanie, Joyce Lamin, Christopher Davis, Gédéon Ngiala Bongo, Michael Dawson, Rashid Ansumana, Dianah Sondufu, Emma C. Thomson, Foday Sahr, Elisabeth Fichet-Calvet

**Affiliations:** 1 Mercy Hospital Research Laboratory, Bo, Sierra Leone; jagbuanie@gmail.com (J.B.); joycelamin@gmail.com (J.L.); mikedawsonfoday72@gmail.com (M.D.); rashidansumana@gmail.com (R.A.); dsondufu@gmail.com (D.S.); 2 Department of Virology, Bernhard Nocht Institute for Tropical Medicine, 20359 Hamburg, Germany; gedeonbongo@gmail.com; 3 Centre for Virus Research, University of Glasgow, Glasgow G61 1QH, UK; Chris.Davis@glasgow.ac.uk (C.D.); Emma.Thomson@glasgow.ac.uk (E.C.T.); 4 College of Medicine and Allied Health Sciences, University of Sierra Leone, Freetown PMB 595, Sierra Leone; fsahr65@gmail.com

**Keywords:** Lassa virus, seroprevalence, *Mastomys natalensis*, abundance, phylogenetics, Bo, Sierra Leone, West Africa

## Abstract

**Simple Summary:**

Lassa fever is a viral hemorrhagic fever caused by the Lassa virus (LASV). It is a deadly rodent-borne zoonosis with outbreaks occurring mostly in Sierra Leone, Guinea, Liberia, and Nigeria, in West Africa. In Sierra Leone, surveillance activities of LASV focus mainly on the Kenema area in the eastern region, known to be the epicentre. Little is known about the presence of the virus in the Bo area, where *Mastomys natalensis* and *Rattus rattus* share habitats with humans. Our study investigated the circulation and phylogeny of new LASV strains and virus seroprevalence in rodent populations of villages in Bo district. Information provided here will be of great importance in prioritizing areas for Lassa fever surveillance and preventive measures to mitigate future outbreaks. Our rodent longitudinal survey carried out over two years (2014–2016) identified *Mastomys natalensis* as the most prevalent species. While seropositive small mammals were found in every village, the four *Mastomys natalensis* rodents that tested PCR-positive for Lassa virus were found in only two villages. Phylogenetic analysis showed that these sequences belong to the Sierra Leonean clade, within lineage IV. In conclusion, LASV is present, with low circulation, in small mammals in rural settings around Bo.

**Abstract:**

Lassa fever is a viral hemorrhagic fever caused by the Lassa virus LASV, which was first isolated in the rodent *Mastomys natalensis* in 1974 in Kenema, Sierra Leone. As little is known about the abundance and the presence of LASV in rodents living in the Bo area, we carried out a small mammal longitudinal population survey. A standardized trapping session was performed in various habitats and seasons in six villages over two years (2014–2016) and samples collected were tested for arenavirus IgG and LASV. A Bayesian phylogenetic analysis was performed on sequences identified by PCR. A total of 1490 small mammals were collected, and 16 rodent species were identified, with *M. natalensis* (355, 24%) found to be the most prevalent species. Forty-one (2.8%) samples were IgG positive, and 31 of these were trapped in homes and 10 in surrounding vegetation. Twenty-nine of 41 seropositive rodents were *M. natalensis.* We detected four LASV by PCR in two villages, all found in *M. natalensis*. Phylogenetic analysis showed that the sequences were distributed within the Sierra Leonean clade within lineage IV, distinguishing a Bo sub-clade older than a Kenema sub-clade. Compared to other settings, we found a low abundance of *M. natalensis* and a low circulation of LASV in rodents in villages around Bo district.

## 1. Introduction

Lassa virus (LASV) is an Arenavirus belonging to the family Arenaviridae, which causes a viral haemorrhagic fever (VHF) known as Lassa Fever (LF). It is a deadly rodent-borne zoonosis with a wide range (mild to severe) of clinical manifestations, including fever, headache, muscle ache, chest pain, sore throat, and gastrointestinal symptoms. The onset of haemorrhage and neurological complications are associated with high mortality. Similarities in clinical presentation with other tropical diseases like malaria and typhoid fever in endemic areas often creates delayed or missed diagnosis within healthcare settings [1,2,3].

The virus is endemic in West Africa with outbreaks occurring mostly in the Mano River Union (MRU) region (Guinea, Sierra Leone, and Liberia) and Nigeria, where the virus was discovered in 1969. It is estimated that the virus infects as many as 200,000–300,000 persons per year with 5000–10,000 fatalities [4]. While the case fatality is low (1–2%), in endemic communities, it can reach 50% in hospitalized patients during outbreaks. Models suggest that approximately 80% of both Sierra Leone and Liberia, 50% of Guinea, 40% of Nigeria, 30% each of Cote d’Ivoire, Togo, and Benin, and 10% of Ghana are LF risk areas, with 200 million people at risk [5].

The Natal multimammate mouse *Mastomys natalensis* was long considered to be the only host reservoir for the virus. It is widely distributed across west and other tropical African countries [6,7]. *M. natalensis* is predominately found in dwellings, especially during the dry season in rural communities [8]. However, recent studies have implicated *Mastomys erythroleucus* in both Nigeria and Guinea, and *Hylomyscus pamfi* in Nigeria as alternative hosts [9]. Humans most likely become infected by exposure to objects contaminated with rodent droppings, consuming rodents infected with the virus, and possibly by inhaling aerosols containing the virus [10,11,12]. Human to human transmission occurs both in the community and in healthcare settings [13].

Sierra Leone is denoted as an LF endemic country, with thousands of cases recorded since the 1970s in the east of the country. Studies have confirmed the spread of the virus within the country for thirty years [3,14,15]. The seroprevalence in certain parts of the human population in Sierra Leone ranges between 8% and 40% [4,16]. As LASV surveillance activities have predominantly focussed on Kenema District, little is known about LASV circulation in Bo, where *M. natalensis* and the black rat *Rattus rattus* co-habit in human dwellings [17]. Nevertheless, information on the circulation of LASV in Bo District would be of great importance in prioritizing areas for Lassa fever surveillance, and resource allocation for measures like rodent control to prevent human infection and future outbreaks. This study was therefore designed to explore the circulation of LASV in villages in Bo District, estimate the LASV prevalence in rodents, investigate the phylogeny of new LASV strains, and to estimate the prevalence of LASV antibodies in the rodent population.

## 2. Materials and Methods

**Figure 1:**
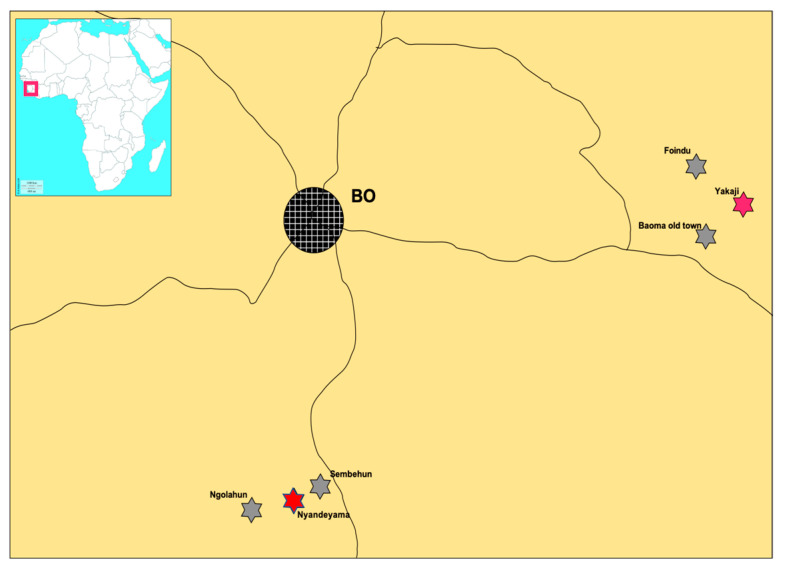
Location of the six villages in Bo district; Baoma Old Town (7°56′42.10″ N, 11°25′53.26″ W), Foindu (8°0′20.05″ N, 11°25′40.23″ W), Yakaji (7°58′23.05″ N, 11°23′44.52″ W), Ngolahun (7°45′25.32″ N, 11°46′12.68″ W), Nyandeyama (7°45′43.98″ N, 11°44′33.23″ W) and Sembehun (7°46′18.31″ N, 11°43′34.22″ W).

The study was conducted in six villages in Bo District with 600–1000 inhabitants each, at a distance of over 30 km from Bo city. We avoided Bo city and suburban areas because of possible colonization by the domestic mice *Mus musculus* and black rats *Rattus rattus*. The predominant occupation of inhabitants of Foindu, Yakaji, and Baoma Old Town at the east of Bo district, near the border with Kenema district is farming. In Sembehun, Nyandeyama, and Ngolahun in the south of Bo district, the local economy is dependent on mining (Figure 1).

### 2.1. Small Mammal Sampling

All villages were sampled for eight consecutive trapping sessions, which started in April 2014 and lasted until February 2016. Following a stratified methodology, habitats sampled for trapping in each village included houses, surrounding fields, orchards, and forests. Between 200–260 live rodent Sherman traps (H.B. Sherman Trap Co., Tallahassee, FL, USA) were set per village per night in the various habitats. All sampled houses were on a straight line; 2 traps were set per room and a maximum of 6 traps were placed per household with up to 3 rooms. In the surrounding fields, orchards and forests, traps were placed 5 m apart in transects of approximately 100 m. The traps were set in the evening hours, were baited with a combination of groundnuts, dry fish, and wheat flour and collected the next morning for three consecutive trapping nights [8]. Trapping continued every 3 months with each session lasting for three nights per village, totalling 33,354 trapping nights.

Following published guidelines [18,19], trapped rodents were first anaesthetized using isoflurane. Their weights were recorded and sexes were determined by inspecting the scrotum and nipple for both males and females, respectively. The length from head to tail, tail length, and the length of hindfoot, and ear were recorded. Based on the morphology, a preliminary species classification was carried out in the field using a published taxonomic key [20]. The identification of *Mastomys* species and those with uncertain identifications were further confirmed using molecular methods, [21,22]. A cardiac puncture was done, and some of blood was dripped on filter paper (SEROBUVARD—LABOCEA, Ploufragan, France) and air-dried, while the remaining blood was aliquoted into collection tubes.

### 2.2. Serology

Immunofluorescence slides containing LASV Bantou289 and AV strains were prepared in the Biosafety Level 4 laboratory at the Bernhard Nocht Institute for Tropical Medicine. Two sets of slides were used for testing, namely regular slides with cells infected on both top and bottom rows were used to test all samples for arenavirus IgG, and mixed slides with infected top row cells and uninfected bottom row cells, were used to recheck samples with uncertain signal of arenavirus IgG. The samples were inactivated in 1:20 dilutions in phosphate-buffered saline (PBS) containing 1% Triton. In cases of missing samples, dried blood spots from filter papers were eluted in a 300 µL solution of 1× PBS containing a 0.2% concentration of ammonium, 2.5 µL of a 25% NH_3_ solution [23]. About 7 µL of the sera or dried blood elutes were dripped on the cells and incubated at 37 °C. Application of anti-mouse immunoglobulin G (IgG) fluorescein isothiocyanate to the cell enhanced binding of IgG, and was detected with fluorescence microscopy. Cells that appeared brightly fluorescent under the microscope were considered positive and confirmed by blinded verification by a second person [24].

### 2.3. RT-PCR Workflow and Sequencing

DNA was extracted from the liver using the QIAGEN blood & tissue kit. PCR targeting cytochrome b was done for confirmation of *Mastomys* species [21,22]. RNA from whole blood and filter paper were extracted first by pooling three samples, using the QIAamp Viral RNA kit (QIAGEN, Hilden, Germany). These were tested by RT-PCR using a OneStep RT-PCR kit (QIAGEN) with two Lassa specific assays, the first with a fragment length of 300 base pair (bp) within the glycoprotein (GP) region of the S-segment, and a Pan-arena assay with a fragment length of 395 bp within the LASV polymerase region [25,26]. Pools positive for any of the assays were re-extracted individually, and tested again using both diagnostic assays to confirm positive individuals. We completed testing of both the GP and the nucleoprotein (NP) genes of positive samples to obtain PCR product for sequencing using several sets of primers [27,28]. Nested and semi-nested PCRs were carried out to generate suitable DNA fragments for sequencing in instances where the DNA bands were very weak or absent. Some gaps in the viral strains were closed by using specific primers designed in this study and listed in Supplementary Table S1.

Sanger sequencing: Prior to sequencing, amplicons obtained by conventional RT-PCR were purified with “NucleoSpin Gel and PCR Clean-up” (MACHEREY-NAGEL) and sent for Sanger sequencing to a private service (LGC, Berlin, Germany, Biosearch Technologies).

Metagenomic next-generation sequencing (NGS): A second RNA extraction was carried out on positive samples, replacing carrier RNA with linear acrylamide (Invitrogen). Real-time PCR was performed with the RealStar Lassa Virus RT-PCR Kit 2.0 (altona Diagnostics, Hamburg, Germany) to obtain Cycle threshold (Ct) values. RNA extracts were sent to the MRC-University of Glasgow Centre for Virus Research for NGS. The protocols were performed as described [29,30]. Briefly, the first-strand cDNA synthesis was prepared by following the recommendations and guidelines for SuperScript III First-Strand Synthesis System for RT-PCR (Invitrogen, life technologies, Waltham, MA, USA), and second-strand cDNA synthesis using the NEBNext kit (New England BioLabs, Ipswich, MA, USA). Construction of libraries for the Illumina MiSeq platform was done using a KAPA LTP Library preparation kit (KAPA Biosystems, Wilmington, MA, USA). Starting with fragmented dsDNA, end repair and A-tailing protocols were performed and resulting cDNA purified with 0.9× AMPure XP (Beckman Coulter, Brea, CA, USA) magnetic beads (Beckman Coulter). Adapter ligated, and libraries amplified (using KAPA HiFi HotStart ReadMix and primer mix) with the recommended PCR profile, and again purified amplicons using 0.9/1 AMPure XP magnetic beads to sample ratio, incubated at room temperature for 5 min, and eluted in a final volume of 13 µL elution buffer (10 mM Tris-HCl, pH 8.0–8.5). We quantified libraries with Qubit and performed Tape-station (Agilent 2200) analysis [30].

Fastq files from Illumina sequencing were first processed via de novo assembly and reference mapped against whole-genome LASV using Tanoti. (http://www.bioinformatics.cvr.ac.uk/tanoti.php) [30]. Sequences generated with both NGS and Sanger Sequencing techniques were assembled and aligned using MacVector v17.5.4 software (2020 Mac Vector, Inc., Apex, NC, USA). The program FindModel (http://www.hiv.lanl.gov/content/sequence/findmodel/findmodel.html) identified the general time-reversible model of sequence evolution with a gamma distribution of among-site nucleotide substitution rate variation (GTR+gamma) as the substitution model that best describes the data set. The phylogeny was inferred by the Bayesian Markov chain Monte Carlo (MCMC) method implemented in BEAST software [31]. The analysis was performed using various assumptions and model settings to evaluate the robustness of the estimations inferred from the models. The following settings were used: GTR+G4 (with 3-partition of codon positions 1, 2, and 3), strict clock, and constant size coalescent tree prior setting. MCMC chains were run for 10 million iterations and sampled every 10,000 states to obtain an effective sample size (ESS) above 200 for all the parameters. Models were compared using a posterior simulation-based analogue of Akaike’s information criterion through MCMC (AICM) [32]. The comparison was conducted in Tracer v1.7.1 using log files containing 1001 states with a 10% burn-in. We ran TreeAnnotator v1.10.4 with the Maximum clade credibility tree option to obtain a single target tree information produced by BEAST. Tree graphics were manually adjusted in FigTree v1.4.4 software (http://tree.bio.ed.ac.uk/software/figtree/).

### 2.4. Statistical Analysis

The analysis focused on *M. natalensis* abundance (TS = mean trapping success; total number of rodents trapped)/(total trapping nights × 100) and LASV serostatus. TS was analyzed by analysis of variance (ANOVA), exploring the significance of differences between villages, seasons, habitats, and livelihood activities. In the model, TS was entered as the outcome variable, and villages (six levels: Baoma, Foindu, Ngolahun, Sembehun, Nyandeyama, and Yakaji), habitat (two levels: inside houses and outside vegetation), season (four levels: start of the rainy season, rainy season, end of the rainy season and dry season), and livelihood activities (two-levels: farming and mining), as predictor variables. Logistic regression analysis was performed to determine any influence of variables on LASV serostatus of *M. natalensis*. In the model, a binary variable (IgG-positive = 1, IgG-negative = 0) was used as the dependent, and village, season, habitat and livelihood activity as the independent variables. Analyses were performed using Stata/IC 15.1 software (StataCorp 4905 Lakeway Dr, College Station, TX, USA), with a 5% significance level.

## 3. Results

A total of 1490 small mammals were collected in 33,354 trapping nights for all eight trapping sessions during our field survey. There were 16 small mammal species morphologically identified, of which 510 samples were molecularly confirmed by cytochrome b-based PCR. The most commonly sampled species was *M. natalensis* with an overall prevalence of 23.8% (357 in 1490), followed by *Praomys rostratus* (23.2%, 345 in 1490) and *R. rattus* (17.5%, 261 in 1490). *Mastomys erythroleucus* was quite rare in the area (0.9%, 14 in 1490). Six out of 16 small mammal species trapped were commensal rodents, representing 40% (599 in 1490) of all rodents trapped (Table 1).

### 3.1. Abundance of M. natalensis

**Figure 2:**
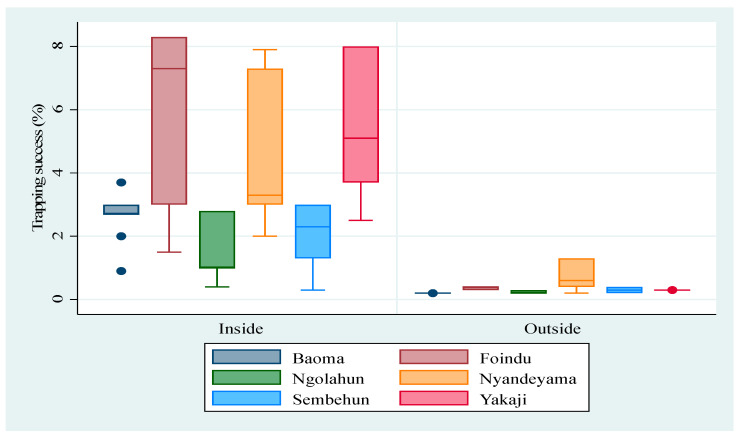
Mean trapping success (in %) of *M. natalensis* by village (Baoma, Foindu, Ngolahun, Nyandeyama, Sembehun, and Yakaji) and by habitat (inside and outside).

**Figure 3:**
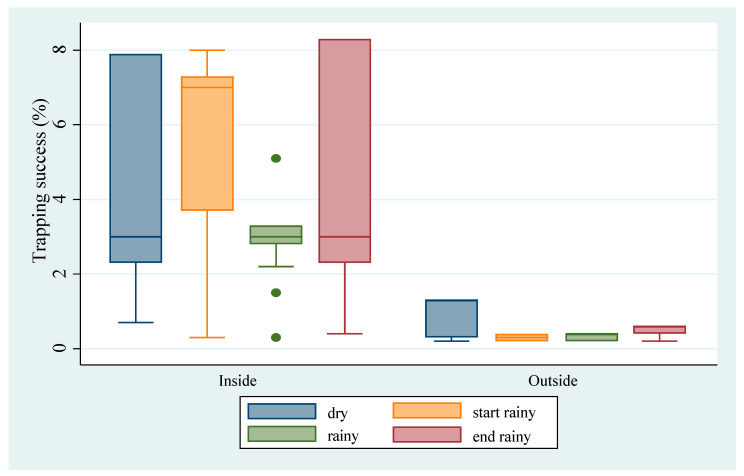
Mean trapping success (in %) of *M. natalensis* by village, season (dry, start rainy, rainy, and end rainy) and by habitat (inside and outside).

Examining the abundance of *M. natalensis* across the six villages, the ANOVA model showed a significant difference in mean TS between villages (*p* < 0.0001) with Foindu (TS = 4.31), Nyandeyama (TS = 3.98), and Yakaji (TS = 4.06) being significantly higher (*p* < 0.0001) than Baoma (TS = 2.57), Ngolahun (TS = 0.67), and Sembehun (TS = 1.25) (Figure 2). Of a total 357 *M. natalensis* trapped, about 92% (328 in 357, TS = 2.72) occurred inside houses, which was significantly higher (*p* < 0.0001) than the 8% (29 in 357, TS = 0.14) that were detected in surrounding vegetation (Figure 2 and Figure 3). To assess the seasonality of abundance of *M. natalensis*, data for corresponding sessions were merged (for example, data for April 2014 and April 2015 were merged) and the analysis focused on only *M. natalensis* trapped inside houses. We observed a significant seasonal variation (*p* < 0.0001), with about 22% (73 in 328, TS = 2.2) occurring in the dry season (February), 40% (130 in 328, TS = 3.7) at the start of the rainy season (April), 14% (46 in 328, TS = 2.1) in the rainy season (July) and 24% (79 in 328, TS = 2.5) at the end of the rainy season (October) (Figure 3). We also observed a significant variation of livelihood activities (*p* < 0.0001), with trapping success being higher in farming communities (58%, 207 in 357, TS = 3.6) than in mining communities (42%, 150 in 357, TS = 2.0).

### 3.2. Serology

Of the 1490 total small mammals captured, 144 sera were not collected. We validated the use of dry blood spot eluates by testing five arenavirus IgG positive sera in parallel with the eluates. The validation gave a sensitivity of 80% of using eluates, as 4 of the 5 elutes tested positive. The method was therefore used to test for arenavirus IgG for small mammals with missing sera but available dried blood spots. We tested the serostatus for a total of 1473 small mammals, using 1346 sera and 127 dried blood spot eluates (17 missing sera and dried blood spots). In total, 41 small mammals tested seropositive for arenavirus IgG (40 sera and one dried blood spot eluate), resulting in an overall seroprevalence of 2.8%. Rodent species that tested positive for arenavirus IgG antibodies included 29 *M. natalensis*, five *P. rostratus*, two *M. erythroleucus*, one *R. rattus,* one *L. sikapusi,* and one *M. edwardsi*. These were distributed in all four trapping seasons. Twenty-nine seropositive *M. natalensis* were found in the six villages, with Nyandeyama (*n* = 12) recording most, followed by Yakaji and Baoma with five each, three in Sembehun, and two each in Foindu and Ngolahun. The seropositive non–*M. natalensis* species were found in all villages except Yakaji, where all five seropositive rodents were *M. natalensis* (Table 2). Of the total 41 arenavirus seropositive small mammals, 31 were trapped inside houses including only three non-*M. natalensis,* whiles 10 other seropositive mammals, including one *M. natalensis* were trapped in the surrounding vegetation. Logistic regression analysis found that the variables village (*p* = 0.11), habitat (*p* = 0.31), season (*p* = 0.8629), and livelihood activities (*p* = 0.094) were not significantly associated with arenavirus serostatus.

### 3.3. LASV Testing

A total of four RT-PCR LASV positive rodents were found in two of the six villages, with three in Yakaji (YAK) and one in Nyandeyama (NYA). All LASV positive rodents belonged to *M. natalensis* and were all trapped inside houses during the first trapping session in April 2014. The sequences are deposited in GenBank under the accession numbers “MW030681–MW030684” for *M. natalensis,* and “MW039388–MW039391” for LASV (Supplementary Table S2).

### 3.4. Phylogenetic Analysis

**Figure 4:**
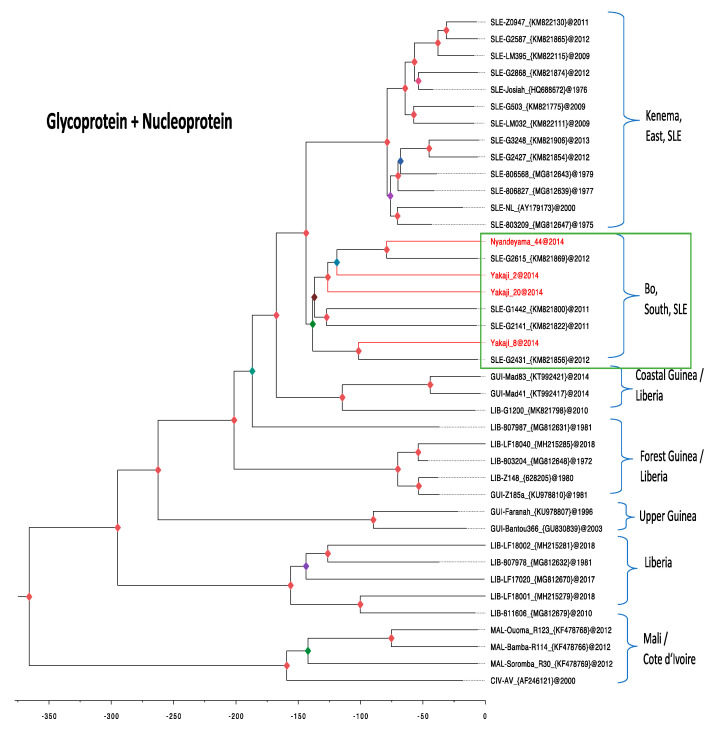
Maximum clade credibility tree of LASV glycoprotein and nucleoprotein. The analysis includes sequences generated in the study (colored red) and those available in GenBank for public use (colored black from Sierra Leone (SLE), Guinea (GUI), Liberia, Mali (MAL) and Cote d’Ivoire (CIV). Statistical support of grouping from Bayesian posterior probabilities are coded as follows: <0.50, brown dots; 0.5 to <0.65, green dots; 0.69 to <0.75, blue dots; 0.82 to <0.86, purple dots; >0.95, red dots. Geographical origins are noted on the right of each tree, and scale axis represents the time in years. Country names, strains, GenBank accession numbers and year of isolation are shown in the tip label.

Forty taxa of the LASV genomes were aligned including the four generated in this study, and 36 others (17 from Sierra Leone, five from Guinea, 10 from Liberia, three from Mali, and one from Cote d’Ivoire) available for public use in the GenBank. A tree of coding-complete regions of glycoprotein precursor and nucleoprotein was constructed in a linked model (substitution, clock, and tree). The analysis shows that our new sequences belong to Sierra Leone cluster in the sub-clade within lineage IV (Figure 4). In particular, “YAK 20” clusters with “YAK 2”, “NYA 44”, and a LASV human-derived sequence from Kenema laboratory (posterior value = 1), and “YAK 08” reliably cluster with another human sequence from the same Kenema Laboratory (posterior value = 1). Our tree clearly distinguished between the Bo and Kenema sub-clades because of the high posterior value (node = 1). Further, our analysis shows that the Bo sub-clade emerged about 138 years ago (median age at 95% highest posterior density interval (HPD), 121.4–156.5) years ago. This is more than half a century older the Kenema sub-clade which emerged about 79 years ago (median age at 95% HPD interval, 71.6–86.6 years ago).

## 4. Discussion

### 4.1. Abundance of M. natalensis

Our two year longitudinal survey in Bo District revealed a higher abundance of *M. natalensis* inside houses than in surrounding vegetation. This finding is in line with ecological studies conducted in Upper Guinea, which recorded a 75% occurrence of *M. natalensis* inside houses [8]. This indicates a strong effect of habitat on the abundance of the species. However, we observed a vast difference in the population of *M. natalensis* within the two geographical regions surveyed for all habitats, with a greater proportion in Upper Guinea (53.5%, 601 in 1123) than in this study (23.8%, 357 in 1490). This translates into a TS in homes in the order of 15% in Guinea [8], but only 3% in this study. Since the sampling methods are comparable (Sherman traps, three days of trapping, two traps per room inside), we can estimate that the population of *M. natalensis* is five times less abundant in the villages around Bo than in the villages around Faranah. However, our studies showed that the two main commensal species, *M. natalensis and R. rattus* shared their habitat in homes in the proportions of 54% (326 in 599) and 42% (250 in 599) respectively. The two-year study confirms the results obtained during the first year [17]. In addition, rodent control practices such as poisoning, introduction of cats and traps by some household members of the communities [33] may have contributed to reducing the number of animals in our study areas. Interestingly, rodent control practices in these areas were not intended to prevent LF, but to protect foodstuff and other items in homes from being eaten or destroyed [33]. Furthermore, *R. rattus,* dominant in Baoma, Ngolahun, and Sembehun, may compete with *M. natalensis* for food, thereby chasing *M. natalensis* out of domestic spaces. The sampling technique employed in this study may have actually under-estimated the population of *R. rattus* due to the Sherman traps used, which were of small size, so that larger adult *R. rattus* may have been prevented from entering the traps.

The seasonality of the abundance of *M. natalensis* in homes may have been associated with various human activities. Our studies showed significantly increased abundance (TS = 3.7) inside houses in April (dry season) and decreased (TS = 2.1) in July (rainy season). This finding is consistent with the doubling of *M. natalensis* in homes during the dry seasons, possibly due to a shortage of foodstuff outside and the availability of stored food inside houses [8]. The study targeted two livelihood communities (mining and farming), revealing a significantly higher abundance of *M. natalensis* in the farming villages (TS = 3.6) when compared to mining villages (TS = 2.0). The likely explanation for this difference may reflect a low quantity of harvested foodstuff stored in homes in the mining villages. Moreover, inhabitants of these villages practiced artisanal mining, and based on our observation, clearing of surfaces for mining was far less compared to clearing of surfaces for farming activities, meaning rodent habitats were less disturbed to cause migration of large numbers of these small mammals to human homesteads. This explanation is in keeping with an observed change in rodent abundance inside houses in Guinea motivated by seasonal foodstuff availability for the rodents, and the use of fields for pasture or land clearing for cultivation [8].

### 4.2. Arenavirus Antibodies in the Rodent Community

*M. natalensis* was the small mammal species with the highest seroprevalence (i.e., 29 out of 41 seropositive small mammals). This is however lower (8%, 29 in 355), than the seroprevalence of 27% (108 in 396) in three LASV high-endemic villages in Guinea [34], and 17% (five in 29) in one village (Eguare-Egoro) in Nigeria [35]. The likely explanation for the observed differences is that our study was conducted in areas where *M. natalensis* was less abundant and few rodents were LASV positive. We also believe that the low abundance of the reservoir did not allow us to detect a difference in seroprevalence between villages or between habitats, contrary to what has been observed in Guinea [34]. Although the second *Mastomys* species, *M. erythroleucus*, is rare in this rain forest zone, it is relatively more infected than *M. natalensis* (14.1%, two in 14). Since *M. erythroleucus* was found infected with a LASV strain (Madina Oula) that is not very different and not spatially far (250 km) from the one circulating in Bo, it can be assumed that host transfer will be able to occur over time and that the virus may be able to persist in two rodent species.

Our study confirms the circulation of arenaviruses in species other than *Mastomys*. As in Guinea, Cote d’Ivoire and Nigeria where similar studies have been conducted, species sharing the same habitat as *M. natalensis* are likely to be infected by LASV [34,35,36]. In East Africa, *M. natalensis* is the host of Morogoro virus, another arenavirus non-pathogenic to humans. A laboratory study has shown that excretion lasts 39 days in urine and saliva, and 29 days in faeces, while seroconversion occurred seven days after infection [37]. The time window for excretion of the virus is therefore quite long, which leaves many opportunities for rodents to contaminate their environment and other species. Transmission of viruses may occur when LASV-positive animals excrete the virus in their urine and droppings [38], causing spill over infection to other small mammal species sharing the same habitat when they come into contact with contaminated surfaces whiles searching for food. This indicates their ability to harbour the virus transiently, and points to rodent species that may become new reservoirs after the virus has become established in its new host.

### 4.3. LASV Infection

In our phylogenetic analysis, the strains from Nyandeyama and Yakaji clustered strongly with sequences previously described in humans from the Kenema laboratory. This suggests that these people may have come from Bo District to receive medical treatment at the Kenema hospital. The LASV sequences described in *M. natalensis* from Barlie, a village north of Bo [39] also cluster with our sequences (see additional Figure 1). This suggests that the sub-lineage of Bo is distinct from that of Kenema. It also appears to be older, with an emergence dating back 150 years, whereas that of Kenema is only 75 years old. Surprisingly, our study detected very few positive animals following a trapping effort carried out over two years. Moreover, all four positive animals were trapped in April 2014, at the end of the dry season. This suggests a break in the transmission chain that could lead to the extinction of the viral strain [40]. This prevalence of 1% (one in 100) in Nyandeyama and 5.9% (three in 51) in Yakaji is much lower than that previously observed in Tongo and Panguma regions, located in the Eastern Province of Sierra Leone, where a prevalence of 17% (14 in 82) was found in *M. natalensis* in September [7]. Similarly, in Upper Guinea, the prevalence of LASV in *M. natalensis* ranged from 11.9% (five in 42) in Gbetaya, 14.3% (31 in 217) in Tanganya, to 15.0% (44 in 294) in Bantou [8].

## 5. Conclusions

In conclusion, our findings have provided strong evidence that LASV is circulating in the small mammal population in Bo District. Comparison with the Faranah site in Upper Guinea, where a similar study was conducted 10 years earlier, shows lower reservoir abundance (five times less, 3% vs. 15%), lower seroprevalence (three times less, 8% vs. 27%) and lower virus prevalence (13 times less, 1.1% vs. 14.5%). Bo is certainly considered as a low endemic area compared to Kenema, but human cases are regularly detected as demonstrated by phylogenetic analysis of the combined GP and NP genes of LASV (Bo cluster visible on the tree). It is therefore curious to note that rodent-related parameters that are a priori favorable for the transmission of the virus (high reservoir abundance, high IgG and LASV prevalence in *M. natalensis*) led to an absence of human cases in the Faranah zone, whereas these same rather unfavorable parameters (low abundance, low IgG, and LASV prevalence in *M. natalensis*) led to the occurrence of human cases in the Bo zone. We hypothesize that widespread circulation of the virus in Upper Guinean villages would protect human populations, because people would be infected early in their lives, and such paediatric cases would be easily missed. Those who are infected early in their lives without becoming seriously ill, and those surviving serious illness in childhood, are then immune, perhaps for the rest of their lives. The high IgG prevalence of 62% in children aged six years old in the Faranah region points in that direction [41]. In Bo region, on the other hand, the low-level circulation of the virus could lead to a large proportion of immunologically naive people, who would be among the acute cases reported in adulthood.

Furthermore, the fact that cases are found in Bo but not in Faranah could also be due to differences in the performance of surveillance and case finding activities. Bo is not far from Kenema, and may have profited from their expertise. For a better understanding of these eco-epidemiological mechanisms, further investigations regarding reservoir abundance and the presence of arenaviruses in small mammal populations should be conducted in localities considered hyper-endemic for LASV, such as those surrounding Kenema. These comparative data will serve as baseline information for public health planning and policies for the treatment and control of LF in Sierra Leone.

## Figures and Tables

**Table 1:** Distribution of commensal rodent species by village in Bo District.

Species	Baoma	Foindu	Ngolahun	Nyandeyama	Sembehun	Yakaji	Total
*Crocidura* spp.		1	1	3		2	7
*Hylomyscus simus*						1	1
*Mastomys erythroleucus*	1	1	1	1	1	1	6
*Mastomys natalensis*	57	93	13	86	30	49	328
*Praomys rostratus*	2	2		4	1		9
*Rattus rattus*	61	25	38	55	39	30	248
Total	121	122	53	149	71	83	599
% *Mastomys natalensis*	47	76	25	58	42	59	54
*% Rattus rattus*	50	20	72	37	55	36	41

**Table 2:** Distribution of arenavirus IgG by species and by village in Bo area.

Species	Village						Total
	Baoma	Foindu	Ngolahun	Nyandeyama	Sembehun	Yakaji	
*Crocidura* spp.	42	24	27	54	35	28	0/210
*Dasymys rufulus*			2		4	1	0/7
*Grammomys buntingi*	1		1				0/2
*Graphiurus* sp.	1						0/1
*Hybomys* sp.		3	4	3	1	5	0/16
*Hylomyscus simus*	8	5	2	1	1	6	0/23
*Lemniscomys striatus*	1			1	1	1	0/4
*Lophuromys sikapusi*	1/41	14	33	1/25	31	30	2/174
*Malacomys edwardsi*	1		1/3	4	6	9	1/23
*Mastomys erythroleucus*	4	1	2	2	2/3	2	2/14
*Mastomys natalensis*	5/60	2/96	2/16	12/100	3/34	5/51	29/357
*Nannomys minutoides*	4	3	2	1		1	0/11
*Nannomys setulosus*	7	2	1	3		11	0/24
*Praomys rostratus*	44	1/39	3/21	1/102	48	92	5/346
*Rattus rattus*	1/61	1/26	40	58	42	32	2/261
*Uranomys ruddi*					2		0/2
Total	7/276	4/213	6/154	14/356	5/204	5/270	41/1473 (2.8%)

## Data Availability

The data presented in this study are available in supplementary material.

## Supplementary Materials

The following are available online at https://www.mdpi.com/2079-7737/10/1/28/s1, Figure S1: Maximum clade credibility tree of partial Nucleoprotein (NP) gene (620 nt) of LASV, Table S1: Oligosequence primers and PCR assays used in this study, Table S2: LASV detected and their Accession numbers deposited in GenBank.
