# Supplementary material for: Lassa Virus Circulation in Small Mammal Populations in Bo District, Sierra Leone
Source: Biology (Basel). 2021 Jan 5;10(1):28. doi: 10.3390/biology10010028 (PMC7824740; doi:10.3390/biology10010028)
Supplement: Supplementary file 1 [file biology-10-00028-s001.pdf]

Lassa virus circulation in small mammal populations in Bo district, Sierra Leone

Supplementary information

Table 1. Oligosequence primers used in this study to perform the different PCR assays.

Primers	Oligosequence, 5'→3'	Target gene	Reference
L7	ACC AAT GAC ATG AAA AAT CAT CGT T	Cytochrome b	[1]
H15915	TCT CCA TTT CTG GTT TAC AAG AC		[1]
F-49	CAT TCA TTG ACC TAC CTG CT		[2]
R-505	AGA ATC CCC CTC AAA TTC AC		[2]
F-607	5'-CGG GCT CTA ATA ACC CAA CG		[2]
R-813	TTC TGG TTT GAT ATG GGG AGG T		[2]
LVS36	ACC GGG GAT CCT AGG CAT TT	GPC	[3]
LVS-339-d	GTT CTT TGT GCA GGA (AC)AG GGG CAT (GT)GT CAT		[3]
LVS 612+	ACCTTCATGAGRATGGCTTGGGG		[4]
LVS 1474-	atgCCCATGTGrTTsAGyCTrTG		[4]
OWS 1000-	AGCATGTCACAGAAYTCYTCATCATG		[5]
OWS 700+	TGATTATTCAGAAYACAWCCTGGGA		[5]
OWS 1430-	CCTWYTATGTGYCTATGTGTTGG		[5]
LVSsl 1430-	CCCACAATRTGCCTATGRGTTGG		This study
LVSsl 1682+	CTGCTGCGTCAAACATGATG		This study
LVSsl 1687+	GCGTCAAACATGATGCAGTC		This study
LVS 1607+	GGTGTTGATGTTCTAAASACC	NP	[6]
LVS 1629+	TGTCTCTGGGCAGCACTGCTC		[6]
LVS 2429-	TGTTTGTCTCAGACACTCCYGGTG		[6]
LVS 2535-	GCCTGCATGTTGGATGGTGGC		[6]
LVSsl2535-	GCYTGCATGYTGGATGGTGGTAAC		This study
OWS 2165A+	TCT TCA GGT CTC CCT TCW ATG TCN ATC CAN GT		[5]
OWS 2165B+	TCT TCA GGT CTC CCT TCW ATG TCN ATC CA		[5]
OWS 2170+	CTC CCT TCW ATG TCN ATC CAN GT		[5]
OWS 2840A-	AAY AAY CAG TTT GGG ACN ATG CCA AG		[5]
OWS2840B-	AAY AAY CAG TTT GGG ACN ATG CC		[5]
OWS 2805+	GTC AGG CTT GGC ATT GTC CCA AAC TGR TTR TT		[5]
OWS 2810+	CTT GGC ATT GTC CCA AAC TGR TTR TT		[5]
OWS 3400-	GCG CAC AGT GGA TCC TAG GC		[5]
OWS 3400A-	CGC AGA GTG GAT CCT AGG CTA TTK GAT TGC GC		[5]
			[5]
LVL 3359D_Y+	AGAATCAGTGAAAGGGAAAGCAAYTC	L	[7]
LVL_3359G Y+	AGAATTAGTGAAAGGGAGAGTAAYTC		[7]
LVL 3754A_R-	CACATCATTGGTCCCCATTTACTATGRTC		[7]
LVL 3754D_R-	CACATCATTGGTCCCCATTTACTGTGRTC		[7]

Table 2: Accession numbers of the murine and Lassa virus sequences by village and by date of collection.

			Organism				
			Mastomys natalensis		Lassa virus		
**Label**	**Village**	**Date**	**Cytochrome b**	**Accession No.**	**GP**	**NP**	**Accession No.**
NYA 44	Nyandeyama	28 April 2014	+	MW030681	+	+	MW039388
YAK 02	Yakaji	10 April 2014	+	MW030682	+	+	MW039388
YAK 08	Yakaji	10 April 2014	+	MW030683	+	+	MW039388
YAK 20	Yakaji	11 April 2014	+	MW030684	+	+	MW039388

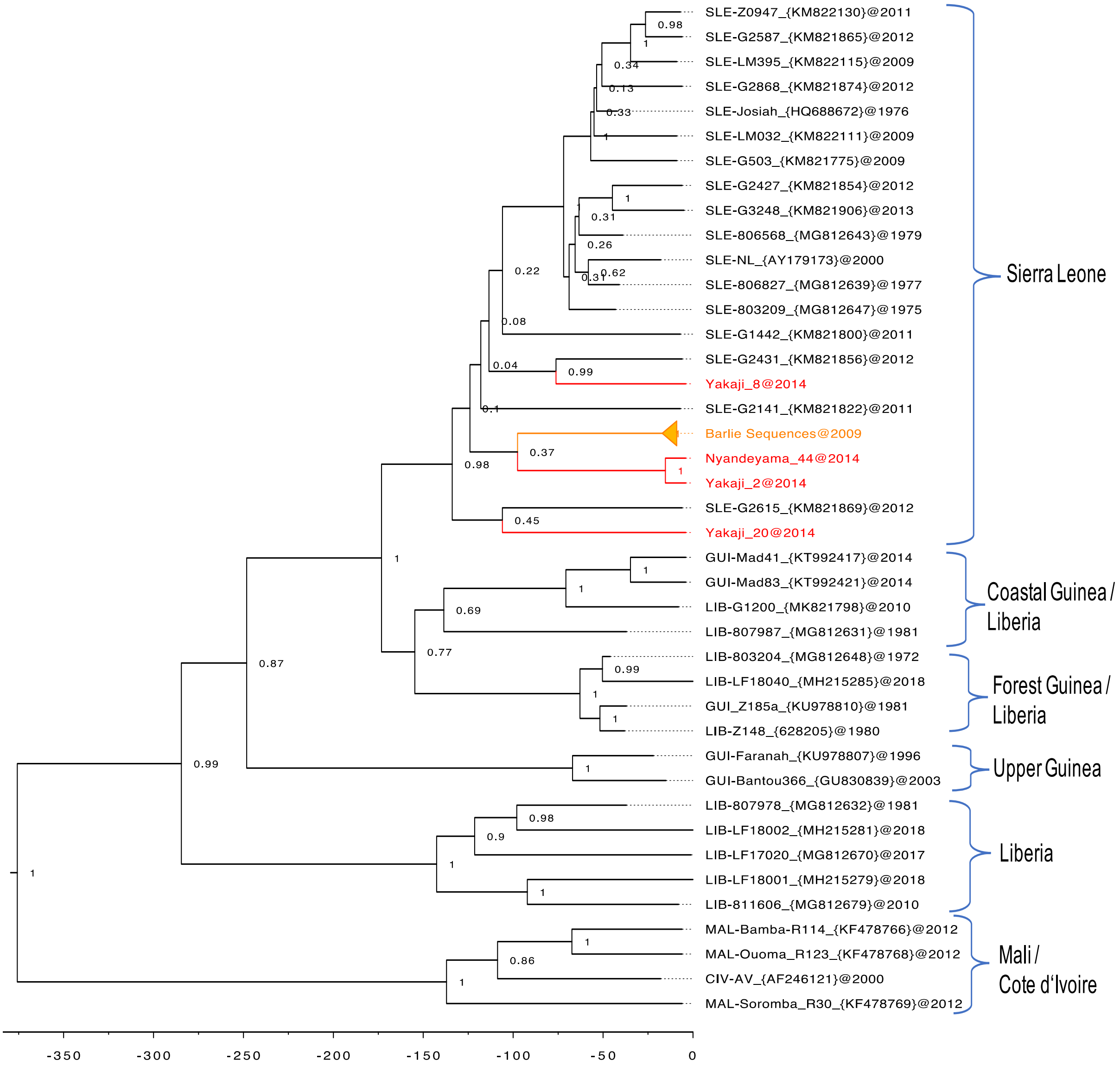

Supplementary figure 1: Maximum clade credibility tree of partial nucleoprotein gene (620 nt) of LASV. The analysis includes sequences generated in this study (coloured red), those from Barlie in Bo district [8](coloured orange) and others from Sierra Leone (SLE), Guinea (GUI), Liberia (LIB), Mali (MAL) and Cote d'Ivoire (CIV). Statistical support of grouping from Bayesian posterior probabilities is indicated at the nodes. Country names, strains, GenBank accession numbers and year of collection are shown on the tips label.

In this figure, we cannot clearly distinguish the Bo sub - clade from that of Kenema due to low posterior values (nodes < 0.5). However, the Sierra Leonean clade is clearly separated from Guinea with high posterior value (node = 1). It might therefore, be certain that all our sequences are also belonging to sub – clade B within the Sierra Leonean clade.

References

1. Ducroz, J.F.; Granjon, L.; Chevret, P.; Duplantier, J.M.; Lombard, M.; Volobouev, V. Characterization of two distinct species of Arvicanthis (Rodentia: Muridae) in West Africa: cytogenetic, molecular and reproductive evidence. *Journal of Zoology* **1997**, *241*, 709-723, doi:10.1111/j.1469-7998.1997.tb05743.x.

2. Lecompte, E.; Brouat, C.; Duplantier, J.-M.; Galan, M.; Granjon, L.; Loiseau, A.; Mouline, K.; Cosson, J.-F. Molecular identification of four cryptic species of Mastomys (Rodentia, Murinae). *Biochemical Systematics and Ecology* **2005**, *33*, 681-689, doi:https://doi.org/10.1016/j.bse.2004.12.015.
3. Olschlager, S.; Lelke, M.; Emmerich, P.; Panning, M.; Drosten, C.; Hass, M.; Asogun, D.; Ehichioya, D.; Omilabu, S.; Gunther, S. Improved detection of Lassa virus by reverse transcription-PCR targeting the 5' region of S RNA. *J Clin Microbiol* **2010**, *48*, 2009-2013.
4. Olayemi, A.; Cadar, D.; Magassouba, N.; Obadare, A.; Kourouma, F.; Oyeyiola, A.; Fasogbon, S.; Igbokwe, J.; Rieger, T.; Bockholt, S., et al. New Hosts of The Lassa Virus. *Sci Rep* **2016**, *6*, 25280, doi:10.1038/srep25280.
5. Ehichioya, D.U.; Hass, M.; Becker-Ziaja, B.; Ehimuan, J.; Asogun, D.A.; Fichet-Calvet, E.; Kleinsteuber, K.; Lelke, M.; ter Meulen, J.; Akpede, G.O., et al. Current molecular epidemiology of Lassa virus in Nigeria. *J Clin Microbiol* **2011**, *49*, 1157-1161, doi:10.1128/JCM.01891-10.
6. Fichet-Calvet, E.; Ölschläger, S.; Strecker, T.; Koivogui, L.; Becker-Ziaja, B.; Camara, A.; Soropogui, B.; Magassouba, N.; Günther, S. Spatial and temporal evolution of Lassa virus in the natural host population in Upper Guinea. *Scientific Reports* **2016**.
7. Vieth, S.; Drosten, C.; Lenz, O.; Vincent, M.; Omilabu, S.; Hass, M.; Becker-Ziaja, B.; ter Meulen, J.; Nichol, S.T.; Schmitz, H., et al. RT-PCR assay for detection of Lassa virus and related Old World arenaviruses targeting the L gene. *Trans R Soc Trop Med Hyg* **2007**, *101*, 1253-1264, doi:10.1016/j.trstmh.2005.03.018.
8. Leski, T.A.; Stockelman, M.G.; Moses, L.M.; Park, M.; Stenger, D.A.; Ansumana, R.; Bausch, D.G.; Lin, B. Sequence variability and geographic distribution of Lassa virus, Sierra Leone. *Emerg. Infect. Dis.* **2015**, *21*, 609-618, doi:10.3201/eid2104.141469.
